# Knowledge, Attitudes, and Practices Regarding Avian Influenza Among Owners of Backyard Flocks — United States, July–December 2025

**DOI:** 10.15585/mmwr.mm7518a2

**Published:** 2026-05-14

**Authors:** Melissa A. Rolfes, Leah Bauck, Beth A. Lipton, Sara F. Margrey, Rebecca A. Campagna, Elizabeth Harker, Colin A. Basler, Courtney M. Dewart, Sascha R. Ellington, Stacy M. Holzbauer, Malia J. Ireland, Jeremy W. Kuo, Christine M. Szablewski, Lizette O. Durand, Carrie Reed

**Affiliations:** ^1^Influenza Division, National Center for Immunization and Respiratory Diseases, CDC; ^2^Minnesota Department of Health; ^3^Washington State Department of Health, Tumwater, Washington; ^4^Ohio Department of Health; ^5^California Department of Public Health; ^6^One Health Office, Office of the Director, National Center for Emerging and Zoonotic Infectious Diseases, CDC; ^7^Division of State and Local Readiness, Office of Readiness and Response, CDC; ^8^Michigan Department of Health & Human Services.

**Summary**

**What is already known about this topic?**

Since 2024, three human influenza A(H5) cases have been reported among people in the U.S. who own backyard birds. Although previous surveys suggest that backyard flock owners are aware of avian influenza, information on knowledge, attitudes, and practices is needed to guide development of education and prevention materials.

**What is added by this report?**

A survey of 638 U.S. backyard flock owners revealed incomplete knowledge about signs and symptoms of avian influenza in humans and birds. Respondents who knew more about avian influenza were more likely to report an intention to use personal protective equipment if they were to interact with potentially infected birds.

**What are the implications for public health practice?**

Education of backyard flock owners by health partners regarding signs and symptoms of avian influenza can help flock owners keep their flocks, themselves, and their families healthy.

## Abstract

Many U.S. households keep backyard bird flocks for their personal food supply or as garden partners. Backyard flocks in the United States have occasionally been infected with avian influenza A viruses, putting flock owners at risk for exposure. During July–December 2025, CDC, in collaboration with state health and agricultural partners, conducted an online survey to learn more about backyard flock owners and their knowledge, attitudes, and practices related to avian influenza. Among 638 respondents who completed the survey, 92% were White (and not Hispanic or Latino), and approximately one half had a graduate or professional degree; a majority kept small, predominantly chicken flocks; and many reported that wild birds could access their flock or the flock’s food or water, which increases the flock’s risk for avian influenza exposure. Although a majority of respondents had heard of avian influenza, approximately one third were unaware of the signs and symptoms of infection in their birds or humans. If they needed to interact with ill or dead birds, a majority of owners knew the recommended precautions to take and indicated willingness to use most, though not all, recommended personal protective equipment. These findings highlight important topics for risk messaging and educational resources so that backyard flock owners are better informed and better able to protect their flocks, themselves, and their families from avian influenza.

## Introduction

Avian influenza A(H5) viruses, commonly referred to as bird flu, circulate among wild waterfowl and seabirds and are causing outbreaks in domestic poultry, dairy cows, and other mammals in the United States; 71 human cases of influenza A(H5) have been reported in the United States since March 2024. Three of these cases, including two deaths (*1**–**4*), occurred among persons who were owners of backyard flocks.

Surveys of U.S. backyard flock owners conducted in 2013 (*5*) and 2018 (*6*), found that a majority of respondents kept small flocks (fewer than 10 birds, primarily chickens) for <5 years. Most respondents were aware of avian influenza, and few reported using personal protective equipment (PPE) during regular interactions with their birds (*6*). To update and build on previous surveys, CDC and state partners conducted a survey among backyard flock owners aimed to assess knowledge of specific signs and symptoms of avian influenza and planned practices if their flock were to become infected with avian influenza viruses. These data might help guide and refine public health messaging to U.S. backyard flock owners.

## Methods

### Data Source

CDC collaborated with state health and agricultural authorities to conduct an anonymous online survey to assess knowledge, attitudes, and practices related to avian influenza.* The survey was hosted in Research Electronic Data Capture (REDCap; Vanderbilt University) and was available in English or Spanish. Survey links were distributed by CDC, state and local public health and agricultural partners, and U.S. Department of Agriculture agricultural extension programs. Links were shared through online platforms including newsletters, social media accounts, and online community forums, as well as with local community groups, at agricultural fairs, and with registered backyard flock owners, depending on the state. The survey was available during July 23–December 3, 2025. This activity was reviewed by CDC, deemed not research, and conducted consistent with applicable federal law and CDC policy.^†^

### Analysis

Survey responses were analyzed in R statistical environment (version 4.5; R Foundation). Respondent overall knowledge of avian influenza was categorized by the number of correct responses to 21 questions about the signs of avian influenza in birds and humans and current events related to avian influenza.^§^ Respondents with 10 or fewer, 11–16, or 17–21 correct answers were classified as having low, moderate, or high knowledge of avian influenza, respectively. Response frequencies among respondent groups were compared using chi-square statistical tests; p-values <0.05 were considered statistically significant.

## Results

### Characteristics of Survey Respondents

Among 747 respondents who started the survey and provided consent for their information to be used, 638 (85%) completed the survey and reported keeping at least one bird in a backyard flock. The 638 respondents who completed the survey did not differ significantly in age or place of residence from the 109 who did not. Respondents lived in 48 U.S. states, 61% lived in a rural setting, 92% reported their race as White (and not Hispanic or Latino), 60% were aged 30–54 years, and 47% reported having a graduate or professional degree (Table). The majority of respondents (58%) reported that at least one person in their household, including themselves, was at increased risk for developing complications of influenza virus infection based on age, including children aged <5 years and adults aged ≥65 years; pregnant women; or persons with certain underlying medical conditions. A majority of respondents were experienced flock owners; 29% had owned their flocks for >10 years, and 48% for 3–10 years. A total of 69% had fewer than 20 birds at the time of survey. Overall, 71% of respondents did not have a veterinarian who they talked to about their flock.

**TABLE:** Characteristics of backyard flock owners and their flocks, by flock setting* — United States, July–December 2025

Characteristic	No. (column %)^†^			
	Total N = 638 (100%)	Urban n = 66 (10%)	Suburban n = 181 (28%)	Rural n = 387 (61%)
**Age group, yrs**				
18–29	**40 (6)**	4 (6)	16 (9)	19 (5)
30–44	**221 (35)**	30 (46)	71 (39)	119 (31)
45–54	**155 (25)**	16 (25)	50 (28)	88 (23)
55–64	**131 (21)**	11 (17)	27 (15)	92 (24)
≥65	**84 (13)**	4 (6)	17 (9)	63 (17)
Unknown	**7**	1	0	6
**U.S. Census Bureau region of residence^§^**				
Northeast	**102 (16)**	0 (0)	44 (24)	58 (15)
South	**122 (19)**	15 (23)	32 (18)	74 (19)
Midwest	**208 (33)**	16 (24)	36 (20)	156 (40)
West	**205 (32)**	35 (53)	69 (38)	98 (25)
Unknown	**1**	0	0	1
**White, not Hispanic or Latino**	**588 (92)**	56 (85)	167 (92)	361 (93)
**Highest education attained in household**				
High school through some college	**118 (19)**	8 (13)	28 (15)	81 (21)
Bachelor's degree	**213 (34)**	23 (36)	58 (32)	130 (34)
Graduate or professional degree	**299 (47)**	33 (52)	95 (52)	170 (45)
Unknown	**8**	2	0	6
**At least one person in household is at high risk for influenza complications**	**372 (58)**	33 (50)	101 (56)	236 (61)
**No. of household members who received the 2024–25 season influenza vaccine**				
All persons in the household	**281 (62)**	38 (70)	93 (65)	150 (60)
Some persons in the household	**152 (34)**	15 (28)	47 (33)	89 (35)
No one in the household	**18 (4)**	1 (2)	4 (3)	13 (5)
Unknown	**187**	12	37	135
**No. of years owning a backyard flock**				
<1	**71 (11)**	12 (18)	28 (16)	30 (8)
1–2	**73 (11)**	12 (18)	25 (14)	36 (9)
3–5	**151 (24)**	19 (29)	45 (25)	85 (22)
6–10	**155 (24)**	11 (17)	46 (26)	97 (25)
>10	**187 (29)**	12 (18)	36 (20)	139 (36)
Unknown	**1**	0	1	0
**Owner has a veterinarian to talk to about the flock**	**184 (29)**	21 (32)	46 (25)	115 (30)
**No. of birds in the flock at time of survey**				
1–4	**76 (13)**	31 (49)	26 (16)	19 (5)
5–19	**322 (56)**	31 (49)	111 (68)	178 (51)
20–49	**116 (20)**	1 (2)	18 (11)	95 (27)
≥50	**65 (11)**	0 (0)	8 (5)	57 (16)
Unknown	**59**	3	18	38
**Type of birds in the flock**				
Chickens only	**472 (74)**	55 (83)	151 (83)	263 (68)
Chickens and ducks or geese	**101 (16)**	6 (9)	17 (9)	77 (20)
Other combinations of birds^¶^	**65 (10)**	5 (8)	13 (7)	47 (12)
**Where the flock spends most of the day**				
Covered run	**303 (47)**	31 (47)	99 (55)	171 (44)
Fenced outdoor area, no cover	**176 (28)**	28 (42)	61 (34)	87 (22)
Free range (minimal or no fencing)	**124 (19)**	5 (8)	14 (8)	103 (27)
Barn, coop, or structure	**28 (4)**	1 (2)	5 (3)	22 (6)
Other	**7 (1)**	1 (2)	2 (1)	4 (1)
Do not know	**0 (0)**	0 (0)	0 (0)	0 (0)
**Flock has some free range**	**355 (56)**	39 (59)	92 (51)	222 (57)
**Can wild birds access the food or water for your backyard flock?**				
Yes	**177 (28)**	21 (32)	46 (26)	109 (28)
Sometimes/Occasionally	**165 (26)**	11 (17)	49 (27)	104 (27)
No	**286 (45)**	33 (50)	82 (46)	169 (44)
Don't know	**7 (1)**	1 (2)	2 (1)	4 (1)
Unknown	**3**	0	2	1

* Four survey respondents who reported their flock setting as “other” are in the total column but were not classified as being in an urban, suburban, or rural setting.

^†^ Percentages were calculated from responses that were not unknown.

^§^ Census Regions and Divisions of the United States

^¶^ Other reported combinations of birds included chickens, ducks, turkey, geese, guinea fowl, quail, peafowl, pigeons, pheasants, and emus.

### Flock Characteristics

Chickens were present in 97% of flocks, and 22% of flocks included more than one type of bird.^¶^ Approximately three fourths (74%) of flocks included only chickens, 16% included chickens with ducks or geese, and 10% included other bird combinations. Approximately one half (47%) of flocks reportedly spent most of the day in a covered run,** and approximately one half (54%) of respondents reported that wild birds could, or sometimes could, access the food or water of the backyard flock.

### Knowledge About Avian Influenza in Birds

Nearly all respondents (94%) had heard of avian influenza or bird flu. A total of 71% knew that humans could be infected with avian influenza by their flock, and 63% knew that humans could be infected by wild birds (Supplementary Table). When asked about current events related to avian influenza, a majority of respondents correctly answered that there have been outbreaks in backyard flocks (89%) and that human cases of avian influenza have been reported in the United States (79%).

**FIGURE 1:**
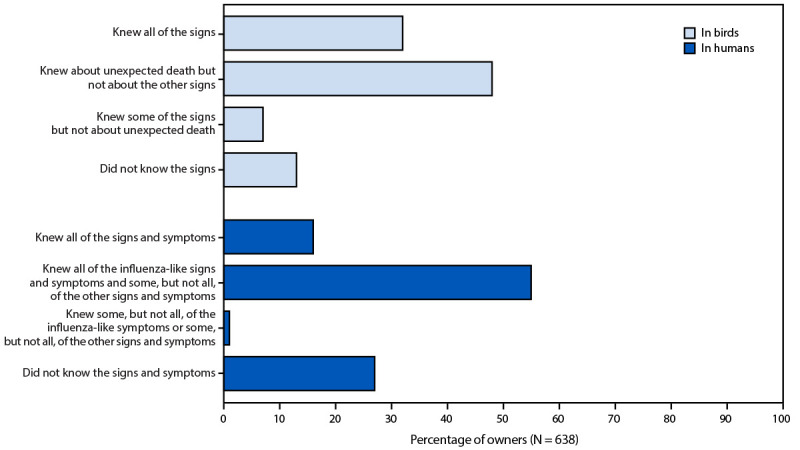
Knowledge of signs and symptoms of avian influenza in birds* and humans^†^ among owners of backyard flocks — United States, July–December 2025 * Signs of avian influenza virus infection in birds include unexpected death, lack of energy and appetite, difficulty breathing, and diarrhea. ^†^ Signs and symptoms of avian influenza virus infection in humans include 1) influenza-like signs and symptoms such as fever, cough, sore throat, runny nose, fatigue, headache, and muscle ache and 2) other symptoms such as pink or watery eyes, diarrhea, and vomiting.

Approximately one third (32%) of respondents correctly identified all signs of avian influenza infection in birds, including sudden or unexpected death, lack of energy or appetite, difficulty breathing, reduced egg production, and diarrhea. Approximately one half (48%) did not identify all signs but did identify unexpected death as a sign; 7% did not identify unexpected death as a sign but identified other signs; and 13% indicated they did not know the signs of infection in birds (Figure 1). If their flock exhibited signs of avian influenza, such as an unexpected death or difficulty breathing, 57% of respondents indicated that they would contact someone, most commonly indicating they would contact a veterinarian, a local department of agriculture, or a local health department.^††^

### Knowledge About Avian Influenza in Humans

Knowledge of signs and symptoms of avian influenza in humans varied: 16% of respondents knew all associated signs and symptoms (including influenza-like symptoms of fever, cough, sore throat, runny nose, fatigue, headache, and muscle ache and other symptoms of pink or watery eyes, diarrhea, and vomiting), 55% correctly identified influenza-like symptoms but not all signs and symptoms, 1% knew some but not all signs and symptoms, and 27% reported not knowing signs and symptoms in humans (Figure 1). Approximately three fourths (77%) of respondents perceived themselves to be at low risk for becoming infected with avian influenza; 28% reported being somewhat or very concerned about avian influenza for their own health.

### Knowledge of Recommended Precautions

**FIGURE 2:**
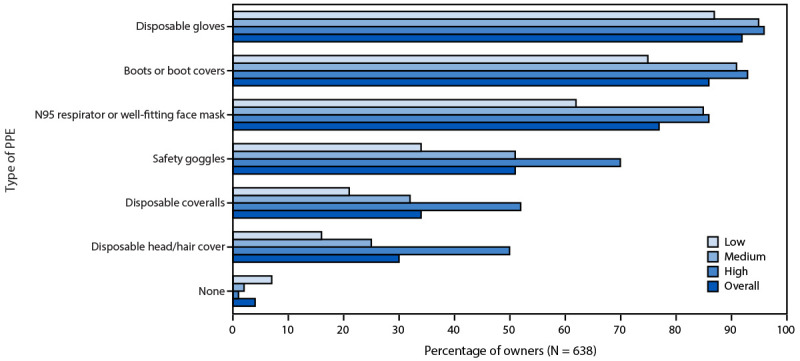
Percentage of backyard flock owners who would use personal protective equipment if their flock exhibited signs of illness, by type of equipment* and by level of knowledge about avian influenza^†^ — United States, July–December 2025 **Abbreviation: **PPE = personal protective equipment. * Chi-square p-values comparing frequencies by level of knowledge are all p<0.01 for each PPE category. ^†^ Knowledge level was categorized based on owner responses to 21 questions related to avian influenza: 17–21 correct answers indicated a high level of knowledge; 11–16 correct answers indicated a moderate level of knowledge; and 10 or fewer correct answers indicated a low level of knowledge.

Approximately 90% of respondents were aware of recommended precautions to take if they suspected avian influenza in their flock, including avoiding touching ill or dead birds, avoiding bringing the ill birds inside their home, avoiding consuming raw or undercooked products from the birds, and using PPE if touching ill or dead birds or their environment. When asked which types of PPE they would use if they needed to touch ill or dead birds or their environment, 92% of respondents reported they would wear disposable gloves, 86% would wear rubber boots or boot covers, 77% would wear an N95 respirator or well-fitting face mask, 51% would wear safety goggles, and 34% would wear disposable coveralls. A total of 3% of respondents reported they would use no PPE. A higher level of knowledge about avian influenza was associated with increased intention to use PPE (Figure 2); all p-values were <0.01 when comparing reported intention of each PPE item by knowledge category.

## Discussion

A majority of surveyed U.S. backyard flock owners had heard about avian influenza, were aware that U.S. backyard flocks have been infected, and knew that human cases of avian influenza have occurred in the United States. However, important gaps in knowledge and prevention practices remain among flock owners, suggesting opportunities for focused public health, animal health, and agricultural outreach.

Many respondents reported that wild birds could come into contact with their flocks, which increases the risk for avian influenza virus transmission. Educational messages should continue to emphasize best practices for keeping flocks healthy by physically separating flocks, feed, and water from wild birds and following other practices suggested in the U.S. Department of Agriculture’s Defend the Flock campaign (*7*), an education program that offers tools and resources for proper practices to protect flocks from illness.

In addition, early recognition of possible avian influenza virus infection in a flock is important for interrupting transmission within the flock and to humans interacting with the birds. Educational messages could emphasize the signs of avian influenza virus infection in domestic or wild birds and provide guidance about contacting a veterinarian or an agricultural or wildlife official for support. Approximately one third (29%) of backyard flock owners reported having a veterinarian. Encouraging flock owners to consider establishing a relationship with a veterinarian might improve early recognition and response to illness in the flock, as well as serve as a resource to help keep birds healthy.

Backyard flock owners should know how to protect themselves from avian influenza. Although most survey respondents reported willingness to use some types of PPE, fewer indicated they would use eye protection or coveralls. Messages to flock owners could highlight reasons to use each piece of recommended PPE, when to use it, and how to use it correctly.

Recent incidences of influenza A(H5) human cases among backyard flock owners in the United States underscore the importance of flock owners knowing the signs and symptoms of possible human A(H5) virus infection. The survey identified limited awareness of nonrespiratory symptoms of avian influenza in humans (such as conjunctivitis, diarrhea, and vomiting) and low perceived personal risk, which could result in delays in seeking health care. Flock owners should be encouraged to seek prompt medical evaluation for any potential symptoms of avian influenza virus infection and report recent bird exposure to health care providers to support timely diagnosis and further infection prevention and control measures.

### Limitations 

The findings in this report are subject to at least two limitations. First, survey respondents represented an online convenience sample of backyard flock owners and therefore might not be representative of all U.S. backyard flock owners. Although no population-based survey data are available regarding the number of persons in the United States who have backyard flocks, data from the 2021 American Housing Survey estimates that 2.7 million households in the United States had pet birds, although backyard flocks could not be differentiated from indoor birds (*8*). Compared with American Housing Survey respondents who had pet birds, survey respondents in this report were more likely to live in a rural setting and have a higher level of education (*9*); therefore, this report might overestimate knowledge of avian influenza among all backyard flock owners. Second, because data were self-reported, responses might have been subject to social desirability bias, which might overestimate the willingness to use PPE or precautions when interacting with ill or dead birds.

### Implications for Public Health Practice

Avian influenza continues to circulate among wild birds in the United States, placing backyard flocks and their owners at risk for infection. Human and animal health partners should continue to educate flock owners about signs and symptoms of avian influenza, appropriate use of PPE, and other practices that can help flock owners keep their flocks, themselves, and their families healthy.

## Footnotes

* The survey included questions on respondent and household demographic characteristics (age, state of residence, setting of premises, highest educational attainment, and race and ethnicity), flock characteristics, other animals on premises, knowledge of avian influenza (current events and signs and symptoms of infection), knowledge and intended use of recommended precautions, and perceptions about risk.

† 45 C.F.R. part 46.102(l)(2), 21 C.F.R. part 56; 42 U.S.C. Sect. 241(d); 5 U.S.C. Sect. 552a; 44 U.S.C. Sect. 3501 et seq.

§ The survey ascertained whether respondents knew about ongoing outbreaks of avian influenza worldwide and in the United States, outbreaks affecting cattle and backyard flocks in the United States, whether there have been human cases in the United States, and signs and symptoms of avian influenza infection in birds and humans.

¶ Respondents reported various other flock combinations (e.g., chickens, ducks, turkeys, geese, guinea fowl, peafowl, pigeons, pheasants, and emus).

** A fully enclosed area with a solid roof, fencing, or wire netting overhead.

†† Participants who reported that they would contact someone if their flock exhibited signs or symptoms of avian influenza were asked a follow-up question about whom they would contact: 43% specified a veterinarian, 40% specified the local agricultural extension, 40% specified the local department of agriculture, 21% specified the local department of health, 12% specified the U.S. Department of Agriculture, and <10% specified “someone else,” the local department of natural resources, CDC, or a physician. Participants could indicate more than one entity in their response.
